# Chromium Substitution Extraction Method for Its Recovery from Chromium-Tanned Leather Waste

**DOI:** 10.3390/ma18010118

**Published:** 2024-12-30

**Authors:** Lesław Świerczek, Paulina Hercel, Izabela Konkol, Ksawery Kuligowski, Adam Cenian

**Affiliations:** 1 Department of Physical Aspects of Ecoenergy, Institute of Fluid-Flow Machinery, Polish Academy of Sciences, Generała Józefa Fiszera 14 Street, 80-231 Gdańsk, Poland; izabela.konkol@imp.gda.pl (I.K.); kkuligowski@imp.gda.pl (K.K.); cenian@imp.gda.pl (A.C.); 2 Renewable Energy Department, Institute of Fluid-Flow Machinery, Polish Academy of Sciences, Generała Józefa Fiszera 14 Street, 80-231 Gdańsk, Poland; paulina.hercel@imp.gda.pl

**Keywords:** leather dechroming, chromium recovery, chromium substitution, chromium extraction, substitutional extraction, collagen recovery, acid extraction, substitutional extraction

## Abstract

The leather industry generates significant amounts of waste, including chromium-tanned leather waste (CTLW), which poses environmental and health hazards due to chromium’s potential toxicity. Efficient management of CTLW is crucial for environmental sustainability and resource recovery. Various methods exist for chromium recovery, including physical, chemical, and biological processes, with chemical methods, particularly substitution extraction using organic acids, showing promising results. This study investigates the use of organic acids for the substitution extraction of chromium from CTLS, with a focus on safety by monitoring the concentration of toxic chromium (VI). It was found that oxalic acid (OA) at a temperature of 43.6 °C and a concentration of 1.34% achieved an extraction efficiency of 63.1% while maintaining minimal hydrolysis at 0.70%. This method offers a sustainable and environmentally friendly approach to chromium extraction from CTLW, addressing a critical need in waste management practices.

## 1. Introduction

Tanned leather, a versatile material in fashion, furniture, and automotive industries, generates significant waste, including chromium sludge, chromium-tanned leather shavings (CTLSs), and chrome leather trimmings [1]. The global leather industry produces around 2140 square kilometers of tanned leather annually [2]. With approximately 15 Mt of leather products produced annually, 16.3% is discarded during finishing.

Due to its environmental and health risks, efficient Cr recovery from CTLW is crucial. Chrome-tanned leather contains 2–4 wt% Cr [3,4], primarily bound to collagen, which enhances the leather’s resistance and impermeability [5].

While Cr(III) salts used in tanning are less harmful, they can cause allergic reactions with prolonged exposure [6]. In contrast, Cr(VI) is highly toxic, causing severe health problems, including skin irritation, respiratory diseases, and organ damage [7,8,9,10]. It has been found that Cr(VI) can penetrate the skin about 10,000-times faster than Cr(III) compounds [10]. Even though Cr(III) is less toxic, its improper management can lead to its conversion to Cr(VI), increasing environmental toxicity [8]. Therefore, efficient recovery of Cr can help reduce its adverse effects and promote sustainable resource use, with potential applications in Cr plating, stainless-steel production, and tannery operations [3,7].

Collagen recovery is equally important due to its wide applications and potential to reduce leather industry waste. Collagen, comprising 80–90 wt% of CTLW [3], serves as a valuable raw material in industries, like food, biopolymers, cosmetics, and pharmaceuticals [11,12,13,14]. However, the efficiency and quality of recovered collagen strongly depend on the processing method of CTLW [15].

The simultaneous recovery of Cr and collagen is key to maximizing resource efficiency and minimizing environmental impacts. While physical methods like incineration and landfilling are cost-effective, they may release hazardous waste [8,16,17]. Biological methods, such as bioremediation, are environmentally friendly but limited by the persistent nature of CTLW [18,19]. Chemical methods are more suitable for CTLW management as they allow for the simultaneous recovery of Cr and collagen. However, this is more challenging than recovering them separately due to issues, like Cr(III) oxidation, wastewater or solid residue generation, partial collagen hydrolysis, and the need for complex processes or auxiliary substances [3,12,20,21,22,23].

Substitution extraction using organic acids or their salts has emerged as a promising method for Cr dechroming due to its environmental benefits, including biodegradable agents, low toxicity, and high Cr recovery rates, while preserving collagen quality [4,24,25,26,27,28]. However, factors like pH, temperature, and acid concentration are critical to balance Cr extraction and collagen preservation [29].

Early studies by D. Brown et al. on Cr recovery from CTLW using substitution extraction explored citric and oxalic acids, achieving 78% recovery at 50 °C in 8 h. The authors noted that temperatures above 60 °C led to significant collagen decomposition and subsequent gelation [30]. Belkacemi et al. [27] achieved 86.9% Cr recovery using sodium oxalate at 60 °C over 10 h. However, collagen hydrolysis was necessary to reduce the Cr content in CTLW from 3.25% to 0.39%. Chen et al. [31] developed a green approach using sodium oxalate combined with high-pressure hydrothermal methods, achieving 95.8% Cr removal with less than 10% collagen loss after 10 h. However, efficiently extracting Cr with acid salts requires lowering the pH and raising the temperature (around 60 °C), which leads to partial collagen protein hydrolysis.

Microwave-assisted extraction (MAE) and ultrasound-assisted extraction (UAE) have also been explored for enhancing Cr removal efficiency. Both methods achieved over 98% Cr recovery, with UAE offering a more efficient alternative by reducing time and water usage [28,32,33]. However, collagen preservation is often not considered in these studies [28,33].

In works where the dual aim of product recovery was studied, it was found that longer extraction times and additional chemicals were necessary to prevent collagen hydrolysis. For example, Malek et al. [34] achieved 95.6% Cr extraction and preserved collagen integrity at room temperature over 72 h. Z. Tian et al. [35] achieved 96% Cr removal using a combination of sulfuric and oxalic acids at 40 °C for 12 h, preserving collagen for biomaterials or cosmetics. L. Wang et al. [29] demonstrated that sodium oxalate could extract up to 98% Cr and recover over 95% collagen under optimized conditions, suggesting the process is driven by intrinsic reactions rather than mass transfer. The authors suggest that Cr(III) in CTLW forms complexes with oxalate ions and dissociates from the collagen matrix without damaging the peptide chain. It was also found that the leaching process was determined by intrinsic reactions rather than mass transfer.

Given the environmental and health risks of TLW and the growing demand for raw materials, there is an urgent need for sustainable management methods. While existing methods often focus separately on Cr recovery or collagen preservation, this study offers a balanced approach, optimizing conditions to minimize collagen degradation while maximizing Cr extraction from CTLS. Various chelating agents, including citric, oxalic, tartaric, ethylenediaminetetraacetic, and formic acid, were compared to assess their effectiveness for Cr extraction. The extraction was conducted under varying temperature and acid concentrations, without pH-adjusting agents, simplifying the process. Collagen degradation was evaluated based on the total nitrogen content (N_tot_) in the extracts, while Cr(VI) concentrations were monitored to avoid secondary contamination. By examining the interaction between organic acids and their ability to extract Cr without compromising collagen integrity, this work presents a novel, one-step method for sustainable CTLS management. The findings significantly contribute to Cr recovery and collagen recycling, ensuring both environmental safety and resource efficiency. This time-efficient method also addresses a critical research gap in the optimal selection of organic acids and extraction parameters, offering a sustainable solution for CTLS management.

## 2. Materials and Methods

### 2.1. Chromium-Tanned Leather Shavingsand Reagents

Chrome-tanned leather shavings (wet-blue shavings) were collected from a car upholstery company in Lower Silesia, Poland. The shavings were dried, ground to a particle size of less than 1 mm, and stored in a sealed container under dry, cool conditions. Organic acids used in extraction were of ACS Reagent Grade quality, while digestion acids (65% HNO_3_ and 35% HCl) were ultra-pure.

### 2.2. Substitution Extraction Experimental Procedure

#### 2.2.1. Extraction Solution Preparation

Substitution extraction studies were conducted with five organic acids: tartaric (TA), oxalic (OA), citric (CA), formic (FA), and ethylenediaminetetraacetic (EDTA). The experiments were split into two stages: the first examined Cr complexation at equal acid radical concentrations, and the second assessed how varying acid concentrations affected Cr removal efficiency.

First, the complexation capacity of individual acids was considered. Due to the low degree of dissociation, organic acids remain partially undissociated in the extraction solution [27,34], which can be generally expressed by Equation (1):

(1) $$HA+H_{2}O\;⇄H_{3}O^{+}+A^{-}$$

where:

$\left[HA\right]$—acid concentration [mol/dm^3^];

$\left[H_{3}O^{+}\right]$—hydronium ion concentration [mol/dm^3^];

$\left[A^{-}\right]$—acid radical ion concentration [mol/dm^3^].


$$\left[A^{-}\right]=\left[H_{3}O^{+}\right]$$

It was assumed that the concentration of dissociated acid radicals of the 5 selected organic acids would remain constant at 0.015 mol/dm^3^. The effective concentration for FA (monoprotic acid) was determined based on Equations (2) and (3):

(2) $$K_{A}=\frac{{\left[A^{-}\right]}^{2}}{C_{a}-\left[A^{-}\right]}\;,$$

(3) $${\left[A^{-}\right]}^{2}+K_{A}\left[A^{-}\right]-K_{A}C_{a}=0$$

where:

$K_{A}$—acid dissociation constant;

$C_{a}$—effective acid concentration [mol/dm^3^].

In the case of TA, OA, CA and EDTA, which are polyprotic acids, their gradual and multistage dissociation in water was considered, but in these calculations, the focus was limited to the second dissociation stage, as the subsequent stages had negligible contributions. The effective concentration of the acid was determined using Equations (4) and (5):

For the first degree of dissociation

$$H_{2}A+H_{2}O\;⇄H_{3}O^{+}+HA^{-}$$

$$K_{A1}=\frac{\left[H_{3}O^{+}\right]\left[HA^{-}\right]}{\left[H_{2}A\right]}$$

(4) $${\left[HA^{-}\right]}^{2}+K_{A}\left[HA^{-}\right]-K_{A}c_{0}=0$$

For the second degree of dissociation

$$HA^{-}+H_{2}O\;⇄H_{3}O^{+}+A^{2-}$$

$$K_{A2}=\frac{\left[H_{3}O^{+}\right]\left[A^{2-}\right]}{\left[HA^{-}\right]}$$

$${\left[A^{2-}\right]}^{2}+K_{A2}\left[A^{2-}\right]+\left[HA^{-}\right]\left(\left[A^{2-}\right]-K_{A2}\right)=0$$

(5) $$C_{a}=\left[HA^{-}\right]+\left[A^{2-}\right]$$

where:

$K_{A1}$—first acid dissociation constant;

$K_{A2}$—second acid dissociation constant;

$C_{a}$—effective acid concentration [mol/dm^3^].

After calculating the appropriate concentrations of the extraction solutions based on the above equations for the first stage of this study, as well as determining the percentage concentration range for the second stage, precise amounts of acids were weighed and dissolved in the required volume of deionized water. Prior to each experiment, a fresh batch of extraction solution was prepared. Table 1 presents a detailed description of the parameters of both series of experiments.

#### 2.2.2. Experimental Procedure 

To address mixing challenges between CTLS and acid solutions, a consistent ratio of parameters was set for all tests. In each experiment, 150 mL of organic acid solution was added to a 250 mL conical flask, placed on a thermostated magnetic stirrer, and heated. After reaching the set temperature, 1 g of dried CTLS was added, and the flask was sealed to prevent evaporation. Samples of the reaction liquid were taken at intervals to study extraction kinetics. After 300 min, the mixture was filtered, and the extract stored at 4 °C for analysis.

Chromium extraction efficiency, both in the study of acid complexing ability and substitution extraction, was determined based on Cr concentration in the extracts. Analyses of N_tot_ content in the filtered extraction mixture were used to determine the proteins hydrolysis degree. Each experiment was repeated three times.

### 2.3. Analytical Methods for Cr and N Content Determination in CTLS 

The total Cr content in the CTLS as well as in the extracts was assessed by the Flame Atomic Absorption Spectroscopy (F-AAS) technique with iCE 3300 AA Spectrometer) (Thermo Fisher Scientific Inc., Waltham, MA, USA). Prior to Cr determination in CTLS, sample was dried and ground to fine powder. Then, about 0.2 g of sample was wet digested in SpeedDigester K-425 equipped with Reflux Setup (BÜCHI Labortechnik AG, Flawil, Switzerland). The sample was digested in a mixture of 65% HNO_3_ and 35% HCl (3:1 *v/v* ratio) for 90 min. The obtained solutions were cooled and filtered through a 0.45 µm syringe filter before analysis. For the acid-Cr extracts, after each substitution extraction, they were decanted, filtered through a 0.45 µm syringe filter, and adequately diluted prior to analysis.

The concentration of Cr(VI), which could oxidize from extracted Cr(III) during the process, was determined using the diphenylcarbazide cuvette test method (HACH LCK 313). Samples prepared in accordance with the methodology were then analyzed by a UV-visible spectrophotometer DR3900 (HACH Lange GmbH, Düsseldorf, Germany).

Total nitrogen in both CTLS and Cr extracts was determined by the Kjeldahl method. Prior to analysis, about 0.5 g of a dry CTLS sample or 5 mL of filtered Cr extract was mineralized for 90 min in 10 mL of 98% H2SO4 with a catalyst with SpeedDigester K-425 Unit (BÜCHI Labortechnik AG, Flawil, Switzerland), according to the manufacturer methodology. After digestion, the samples were steam distilled using the K-350 Distillation Unit (BÜCHI Labortechnik AG, Flawil, Switzerland). Each sample was analyzed in three replicates. Multifunction Meter CX-705 with appropriate electrodes (Elmetron, Zabrze, Poland) was used for all pH measurements.

### 2.4. Optimization of Substitution Extraction Process Using Response Surface Methodology (RSM)

Based on the initial determination of the type of acid with the greatest ability to chelate Cr from the collagen structure, the substitution extraction process was optimized. For this purpose, the RSM was selected as a tool to determine the most favorable process parameters. In the present study, a two-factor and three-level central composite design (CCD) was used for response surface modelling. Chromium extraction yield and N_tot_ loss were selected as the response variables. Process temperature and organic acid solution concentration were chosen as independent variables. A total of 13 experiments were performed to determine the optimum conditions for optimum Cr removal, taking into consideration the interaction among the factors. Minitab 17.0 (Minitab Inc., State College, PA, USA) software was used to analyze the results from experimental plan and fit them into a second-order model through CCD.

### 2.5. Data Predictions and Analysis

For the RSM analysis, the ordinary least squared (OLS) method was employed to obtain the fitted regression models predicting the yield of Cr. Two types of models were fitted to the data—quadratic and linear. The respective models were fitted for results of four acid types: TA, OA, CA, and FA (EDTA was eliminated in initial testing). This model aimed to capture potential non-linearities in the data, providing a more nuanced understanding of the process. Equation (6) describes the quadratic model:

(6) $$Y=\beta_{0}+\beta_{1}X_{1}+\beta_{2}X_{2}+\beta_{3}X_{1}^{2}+\beta_{4}X_{2}^{2}+\beta_{5}X_{1}X_{2}+\varepsilon$$

where Y is the response variable (Cr yield), X_1_ is temperature, and X_2_ is acid concentration. Parameters β_0_–β_5_ are the coefficients estimated by the model and ε is the error term.

The linear model served as a baseline for comparison, assuming a linear relationship between the variables. The linear model is presented in Equation (7):

(7) $$Y=\beta_{0}+\beta_{1}X_{1}+\beta_{2}X_{2}+\varepsilon$$

where *Y* is the Cr yield, *X*_1_ is temperature, *X*_2_ is acid concentration, parameters β_0_–β_2_ are the coefficients estimated by the model, and *ε* is the error term.

Both models were trained using the OLS method, optimizing coefficients to minimize the sum of squared differences between observed and predicted Cr yields. All of the calculations, fitting and graphical processing were performed using Python and libraries: statsmodels, sklearn, seaborn, matplotlib, numpy, and pandas.

## 3. Results and Discussion

### 3.1. Substrate Characterization

Several parameters in the analyzed CTLS exhibited noticeable variability. It was observed that improperly stored samples rapidly dried, resulting in the moisture content of CTLS being the most variable parameter. The CTLS used in this research, characterized by the F-AAS technique, contains a substantial amount of Cr (33.66 ± 0.17 gCr_total_/kg TS), consistent with prior literature reports regarding TLW management [27,36]. CTLS is categorized under 04.01.08 of the European Waste Catalogue; however, despite its elevated Cr content, it does not qualify as hazardous waste [37]. The leather tanning process involves the use of various chemicals to impart specific properties to the leather. Consequently, aside from the high Cr content, CTLS contains various mineral substances, which must be controlled after processing [38]. The second most significant element in the tested waste was nitrogen (16.1% TS), primarily sourced from collagen proteins. Furthermore, the content of total solids (TS) and volatile solids (VS), associated with high collagen content [39], ranged, respectively, between 51.94 ± 0.24% and 91.96 ± 0.20%. A comprehensive chemical characterization of CTLS is presented in Supplementary Table S1 and work presented by K. Mikula et al. [11].

### 3.2. Chromium Complexing Ability by Organic Acids

Despite the higher ability of OH^−^ ions to complex with Cr(III), particularly in alkaline extraction techniques, organic acids were used in complexing ability studies to preserve the structural integrity of collagen. The experiments focused on establishing a consistent concentration of acid radical anions and determining their influence on the efficiency of Cr substitution extraction from CTLS. Notably, increased concentrations of H_3_O^+^ ions have the potential to competitively interact with -COO^−^ residues present in collagen. Thus, in all experiments, the original pH value of the solutions was not changed [35,40].

The concentration of organic acids dissociated radicals in the solution was set at 0.015 mol/dm^3^. Based on the provided equations, the actual concentrations of organic acids were calculated and checked by measuring the pH of the prepared solutions. The calculated and measured values are summarized in Table 2.

**Figure 1:**
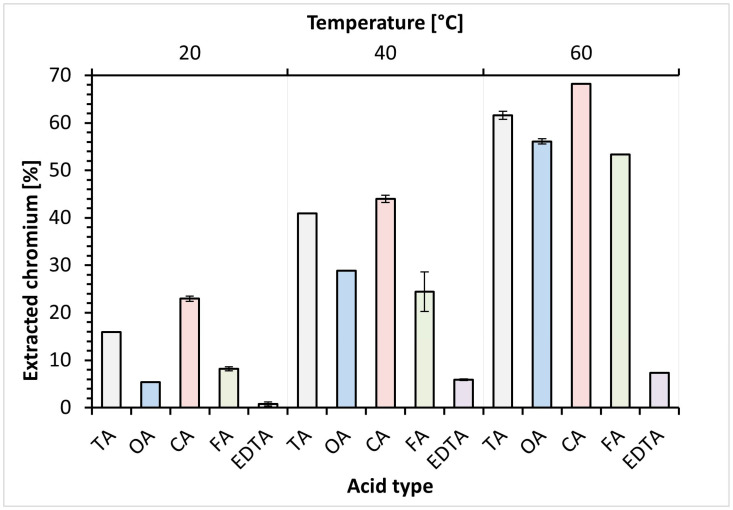
Variation in Cr substitution efficiency based on acid type and temperature. Abbreviations: TA—tartaric acid, OA—oxalic acid, CA—citric acid, FA—formic acid, EDTA—EDTA acid.

The efficiency of Cr extraction via the acid substitution reaction in equimolar concentrations of different organic acid residues at various temperatures is presented in Figure 1. Based on the obtained data, it was found that the process temperature significantly influenced the ability of Cr complexing. In all tests, the Cr extraction efficiency rises with increasing temperature; moreover, in the case of CA, FA and TA, this relationship is linear. It was observed, despite having very good complexing properties, that EDTA had the least significant effect on Cr substitution extraction. The low solubility of EDTA in water (1 g/L at 25 °C) [41], making it impossible to achieve the intended concentration, also had a negative effect on Cr extraction efficiency.

The CA presents the highest ability for Cr ion substitution, with extraction efficiencies of 23.0, 44.0 and 68.2% as the temperature increased. Good complexation ability was also observed for FA, TA and OA, where efficiencies at 60 °C reached 53.3, 61.6, 56.1%, respectively. It is worth noting that the concentrations of CA, FA and TA solutions used for the substitution reaction were an order of magnitude higher than in the case of OA. Nevertheless, the Cr extraction efficiency of OA reached a satisfactory result of 56.1%. This may indicate a high affinity of OA radicals for Cr and the formation of a stable chelate in the solution.

Z. Tian et al. indicated that the complexation capacity of acid radical ions is in the order OA, CA, TA [35]. It is believed that radicals having only one free carboxyl group in the structure have a poor ability to complex Cr bounded to collagen in TLW [27,29]. Due to multistage dissociation, organic acid radicals have additional free carboxyl groups available for metal cation binding. The geometry of these ions also influences Cr chelation [27], while H_3_O^+^ ions generated during acid dissociation notably enhance extraction efficiency [29]. The analysis of test results confirms the influence of all mentioned features in substitution extraction by CA, TA, and OA. The CA exists in various ion forms, such as H_2_C_6_H_5_O_7_^−^, HC_6_H_5_O_7_^2−^, and C_6_H_5_O_7_^3−^, capable of creating stable chelate ligands with Cr, thereby significantly impacting the extraction yield. The TA and OA exhibit a satisfactory ability to substitute and complex Cr due to their two free carboxylic groups and planar compound geometry, making them suitable substances for Cr recovery [27,29].

### 3.3. Chromium Removal Efficiency by Substitution Extraction

Many studies have examined how varying parameters affect Cr removal efficiency, noting that proper selection can enhance extraction by providing more reactive sites for Cr chelation [28,29,35]. Based on previously conducted tests, four acids were chosen as potential chelating agents for effective Cr extraction. The second phase of the research focused on evaluating the impact of temperature and acid concentration on extraction efficiency, addressing discrepancies regarding whether the substitution mechanism is driven by the acid residue molecule or its concentration [27,29].

**Figure 2:**
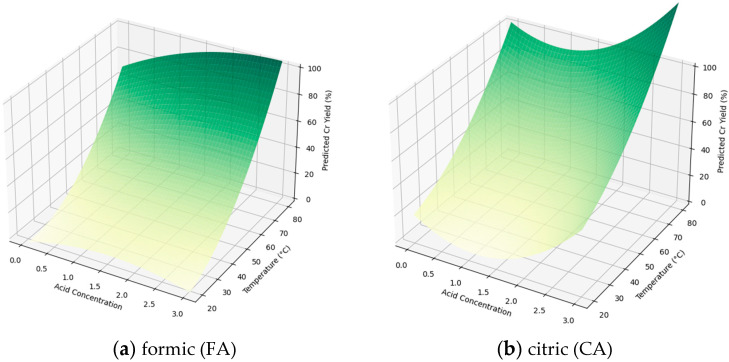

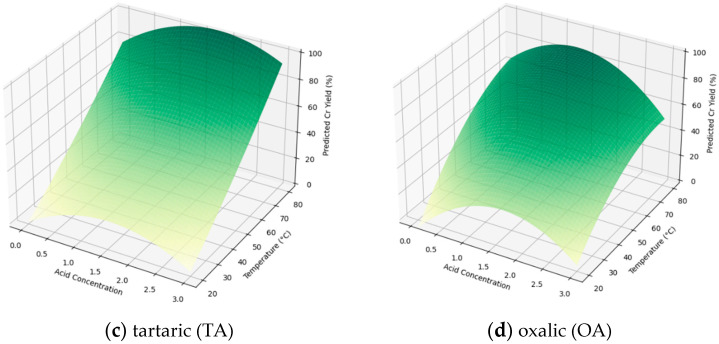
Extraction yield predictions of the quadratic model.

**Figure 3:**
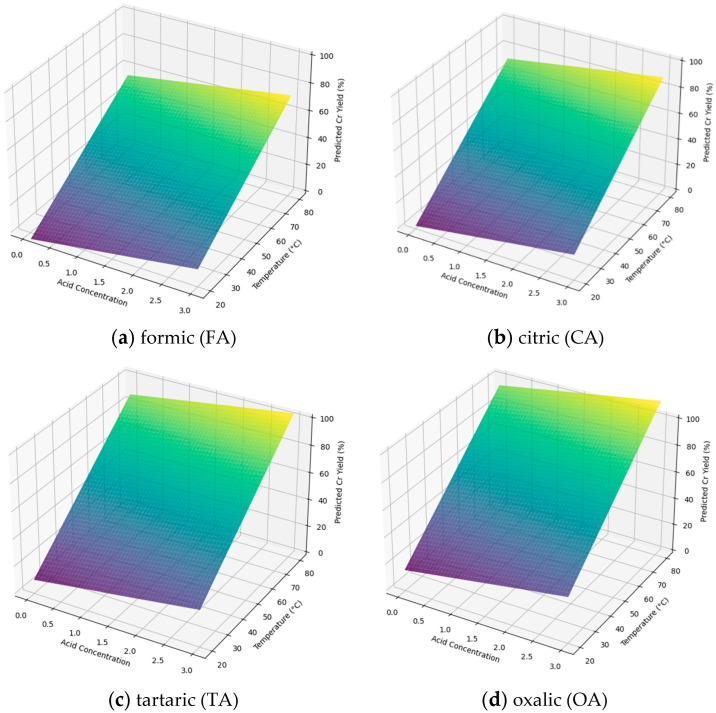
Extraction yield predictions of the linear model.

Figure 2 and Figure 3 illustrate the impact of temperature and acid concentration, showing predictions from both quadratic (yellow-green) and linear models (blue-green) for Cr yield across the experimental parameters. Tables S2–S5 and S6–S9 in the Supplementary Materials summarize coefficients, standard errors, test statistics, and estimates for the models. Tables S10 and S11 present results from fitting both models to the data for all investigated acids.

It was assumed that the relationship between temperature, acid concentration, and Cr extraction yield could be well approximated using a quadratic function. All of the models appear to fit the data well, with high R-squared and adjusted R-squared values. The R-squared values for all the quadratic models are relatively high, at the level of 99% (the only exception is the model for CA, where the value is 96%). Based on the coefficients’ *p*-values (*p* > |t|), it was found that the statistical significance of certain terms differs depending on the acid extraction model used. Therefore, each acid requires a unique extraction model and should, thus, be interpreted independently.

For all of the investigated acids, the quadratic model exhibited strong multicollinearity among the independent variables, with a condition number of 3.4 × 10^4^, which should be taken into consideration during the interpretation of individual coefficients. A high condition number indicates that small changes in the data could lead to large changes in the estimated regression coefficients. This sensitivity can make the interpretation of coefficients less reliable, as their values become less stable. However, if the purpose of the model was solely to predict Cr extraction yield values rather than to ascertain the role of the two independent variables, it can be neglected.

Despite expectations for quadratic models to capture non-linear trends, significant differences in fit between models were not observed, likely due to the narrow range of temperature and concentration, where relationships are nearly linear. For linear models, FA and CA had R-squared values of 92%, indicating poorer fit compared to quadratic models, while the TA and OA models had higher fits (98.6% and 95.4%, respectively) and similar performance to quadratic models. The F-statistic values suggested that linear models fit better for CA (F-statistic = 36 vs. 16) and TA (206 vs. 136) but were comparable for OA (62 vs. 60).

It is worth adding that the developed linear model provides only simplified information regarding the impact of changes in the main extraction parameters on its efficiency. It does not consider all interactions between variables. Additional insights can be derived from the analysis of the quadratic model, which takes more interactions between variables into account. Given the fact that the investigated temperature and acid concentration range is relatively narrow, it can be assumed that the linear model can be sufficient for this analysis; however, using the quadratic model can help in obtaining more precise predictions.

Table 3 lists the results of Cr single-stage substitution extraction, where the yield was over 50%. The research demonstrated that, using all four organic acids, it is possible to reach such a level under specific conditions. The FA proved to be the least effective, necessitating higher extraction parameter values. Nonetheless, FA resulted in the lowest degree of collagen degradation (N_tot_ loss).

It was observed that TA exhibited a higher average Cr extraction yield compared to CA under the same process conditions. Similarly, OA demonstrated favorable extraction rates at 40 °C, achieving a 57% Cr extraction yield in a 1% OA solution with minimal collagen protein hydrolysis.

It was observed that increasing the process temperature significantly enhances the extraction efficiency. However, a simultaneous increase in solution concentration does not always yield positive results. According to predictions from the quadratic model, for TA and OA, a concentration between 0.5 and 2.0% is preferable. Similar trends were seen for FA but only within the 40–50 °C temperature range. Notably, only CA exhibited a synergistic effect with increasing temperature and concentration, resulting in high extraction rates with minimal hydrolysis compared to TA and OA extractions under similar conditions.

Using 2% solutions of TA, OA, and CA at 60 °C, the highest degree of Cr extraction was achieved: 72%, 79%, and 63% respectively. It is worth noting that under such process conditions, significant collagen hydrolysis occurs (see Table 3). According to predictions from the quadratic model for the mentioned acids, temperature may positively impact on Cr extraction yield. However, the risk of complete hydrolysis of collagen proteins should be considered [42].

Based on the analysis of Cr(VI) content in the samples, partial oxidation of Cr(III) to Cr(VI) was observed. Table S16 in the Supplementary Materials presents the concentrations of Cr(VI) in the extracts and the percentage of Cr(VI) relative to the total Cr content in all analyzed samples. The highest Cr(VI) concentrations were recorded for the extraction process at the highest acid solution concentrations and process temperatures, specifically for TA, OA, CA, and FA: 0.20, 0.65, 0.18, and 0.086 mgCr(VI)/L, respectively.

It is important to note that the levels of Cr(VI) found in the solutions comply with the regulatory standards set by the European Union (EU). According to EU regulations, the emission limits for Cr into aquatic environments are set to ensure minimal environmental impact. The maximum permissible concentrations are 1 mg/L Cr(VI) and 5 mg/L for total Cr [43].

It has been demonstrated that, regardless of the parameters, the maximum Cr(VI) content did not exceed 0.6% of the total Cr content (for 0.5% FA at 20 °C), which is mainly due to the low extraction efficiency of Cr(III) and its parallel oxidation to Cr(VI). In the case of extraction with a 2.0% OA solution at 60 °C, the highest concentration of Cr(VI) was recorded at 0.65 mg/L, constituting only 0.35% of the total Cr. Chromium (III) oxidation occurs partially in all cases, where Cr(VI) concentration can be influenced by Cr(III) concentration, used acid type, and the process temperature.

Chelation is a significant factor in the effectiveness of OA for Cr recovery, but additional mechanistic aspects also contribute to its efficacy. Substitution extraction begins with the destabilization and eventual rupture of the collagen-Cr bonds in the leather matrix. The mechanism of this process involves two main aspects. First, organic acids dissociate in water, releasing hydronium ions (H_3_O^+^), which can attack the Cr-collagen bonds in the leather, leading to the release of Cr ions into the solution, which was also observed in many studies about efficient Cr removal by acid solutions [13,24,32,35,44]. However, the acidic environment produced by organic acids can induce hydrolysis of the collagen structure, whereas, in these studies, hydrolysis was limited by process parameters.

The second aspect of the efficient substitution extraction mechanism is connected to the specific character of organic acids and their ability to chelate Cr. Specifically, Cr is connected to the carboxyl side chains of aspartic and glutamic acid on the collagen molecule by two bonds [45], where -OH, -COOH, and -COC are the main functional groups [5,44]. Chelation starts with the exchange of a water molecule and progresses with the attack of ionized carboxylic groups of the chelating agent [27]. In the case of OA, its molecular geometry and multiple carboxyl groups enhance its ability to form stable complexes with Cr ions. The small size and spatial coordination ability of the OA molecule enable it to chelate Cr more effectively compared to other organic acids with similar functional groups, such as TA, which also possesses two free carboxyl groups [27,29].

Once the Cr ions are freed from the collagen, they bind to the acid radical ions, forming stable Cr-oxalate complexes. These complexes have higher solubility compared to other Cr compounds, facilitating easier extraction from the leather matrix. Meanwhile, the H_3_O^+^ cations produced in the acidic environment suppress the dissociation of collagen carboxyl groups, further reducing interaction with the newly created Cr complexes [29,42].

As the extraction progresses, the increase in pH compared to the initial value is particularly noticeable in test series characterized by high Cr yields. This confirms that during extraction, Cr is replaced by H_3_O^+^ cations in the collagen structure, forming stable chelates in the solution. The efficiency of the extraction process is, therefore, significantly influenced by the pH, temperature, degree of acid dissociation, and concentration of the organic acid.

### 3.4. Process Optimization

Taking into account the potential for significant hydrolysis of collagen proteins during Cr recovery, the process was optimized, where the goal was to achieve the highest possible Cr extraction while minimizing the concentration of N_tot_ in the extract, which serves as an indicator of collagen hydrolysis. It was found that OA is the best acid for extraction due to its effectiveness across a wide range of parameters.

The model summary (Table S13 in Supplementary Materials) indicates that the regression model exhibits good predictive ability, as evidenced by the relatively high R-squared and predicted R-squared values. Examining the analysis of variance results (Table S14 in Supplementary Materials), it is evident that the linear component of the model is highly significant (*p*-value = 0.001), suggesting that the linear relationships between the predictors and the dependent variable are statistically meaningful. However, the square component and the two-way interaction component are not statistically significant (*p*-values > 0.05), indicating that these additional terms do not significantly improve the model’s fit.

The results indicate that with each unit increase in temperature, the expected value of the response variable increases by 26.94. Similarly, for each unit increase in concentration, the expected value of the response variable rises by 5.36 (see Table S15 in Supplementary Materials). The coefficient for temperature change (26.94) is greater than that for concentration change (5.36). Furthermore, the T-value for temperature change (16.48) exceeds that for concentration change (3.31). The lower *p*-value for temperature change indicates greater statistical significance compared to concentration change. Therefore, it is reasonable to conclude that temperature change has a more significant effect on the result than concentration change in this model. This is further supported by the Pareto chart (Figure S1 in Supplementary Materials), where temperature factor was represented by the highest standardized effect. The mathematical optimization model is presented according to Formula (8):

(8) $$Extracted\;Cr\;\left[\%\right]=-56.6+2.746T+45.1C-0.01533T^{2}-12.97C^{2}-0.138TC$$

where:

T—temperature [°C];

C—concentration [%].

**Figure 4:**
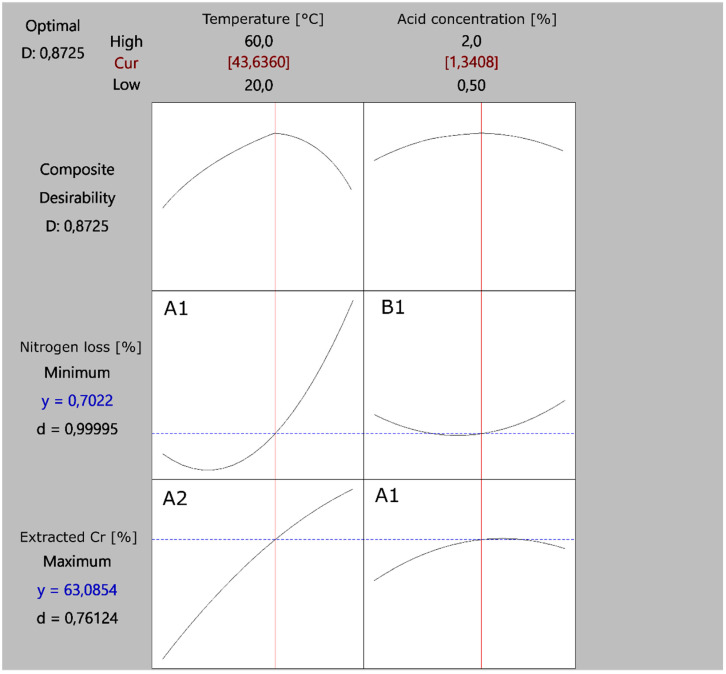
Response optimization plot showing conditions, where “Extracted Cr [%]” is maximized and “Nitrogen loss [%]” is minimized.

Based on the analysis of the obtained results, presented in Figure 4, it was shown that a temperature of 43.6 °C and solution concentration of 1.34% are the optimal parameters for single-stage substitution extraction with OA. These parameters enable an extraction efficiency of 63.1%, while the hydrolysis of TLW is suppressed to only 0.70%.

Since the process temperature has a greater impact on N_tot_ loss compared to acid concentration (see cells A1 and B1 in Figure 4), even a slight reduction in temperature can lead to a significant decrease in collagen decomposition while maintaining a favorable Cr extraction efficiency. Additionally, as previously mentioned, temperature has a greater influence on efficiency than acid concentration (cells A2 and B2). Therefore, increasing the temperature positively affects extraction efficiency but leads to an exponential rise in collagen hydrolysis (cell A1).

**Figure 5:**
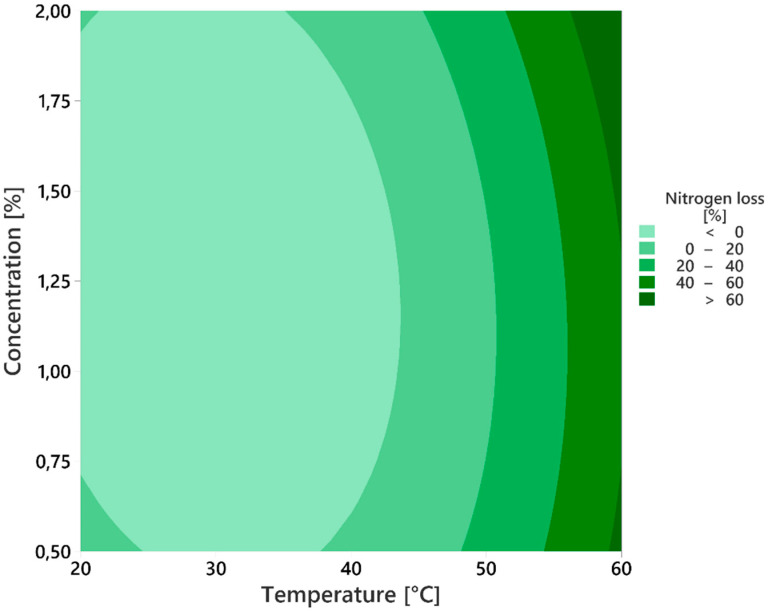
Contour diagram of N_tot_ loss as a function of process temperature and OA concentration.

It was observed that, to completely prevent the hydrolysis of collagen proteins, the process should be conducted within a temperature range of 20 to 40 °C, as illustrated in Figure 5. Additionally, it is evident that an increase in temperature has a more pronounced negative impact on the N_tot_ loss from CTLS compared to an increase in OA concentration. This leads to reduced reagent consumption, resulting in environmental benefits.

The presented substitution extraction method was compared to other similar studies where organic acids were also used, but the dual aim of simultaneous Cr recovery and collagen structure preservation was not always considered. Table 4 presents selected methods for Cr extraction and collagen recovery, along with the method described in this study.

In comparison to other methods, no auxiliary reagents or pH-regulating substances were required in this study. The extraction process involves a temperature increase, but this is lower than that required in other acid-based methods. Additionally, the extraction time was reduced to 5 h, making it the shortest duration (excluding assisted methods). The simplification of the process makes the described method a cost-effective alternative to more complex, multistep approaches.

While many previous studies focus solely on Cr recovery, they often overlook the potential environmental hazards associated with Cr(VI) formation. In this study, a comprehensive evaluation of collagen loss was conducted alongside the monitoring of Cr(III) and Cr(VI) concentrations during the extraction process. This approach can not only ensure a balanced recovery of Cr and collagen but also reduce the risk of secondary contamination by actively monitoring Cr(VI), addressing a critical gap in many existing studies.

It is worth emphasizing that by using additional auxiliary substances, such as urea, which loosens the collagen structure, it is possible to obtain better extraction results [42]. Improved efficiency can also be achieved by conducting substitution extraction in several stages. The additional steps to improve extraction efficiency could serve as future directions for further development of the described method.

## 4. Conclusions

The efficient management of CTLW is crucial due to its environmental and health risks. This study explored the use of organic acids for Cr extraction from CTLS while minimizing collagen degradation. Organic acids showed varying chelating abilities, with OA emerging as a promising agent due to its high affinity for Cr and stable chelate formation, even at low temperatures.

Optimized conditions of 43.6 °C and 1.34% OA solution achieved 63.1% Cr extraction efficiency with minimal collagen loss (0.70%). The process also resulted in partial Cr(III) oxidation to Cr(VI), with Cr(VI) levels remaining well within EU regulatory limits (0.65 mg/L), indicating minimal environmental risk.

This study introduces a novel and sustainable approach to CTLW management, ensuring a balanced recovery of Cr and the preservation of collagen. Future research into process optimization and auxiliary substances may further enhance extraction efficiency and waste management practices.

## Figures and Tables

**Table 1:** Main parameters of substitution extraction experimental procedure.

Experimental Procedure	Organic Acid	Concentration	pH	Temperature [°C]	Stirring Speed [RPM]	Extraction Time [min]
Complexing ability experiments Concentration in [mol/dm^3^]	Tartaric acid (TA)	0.225	1.728	20, 40, 60	500	300
	Oxalic acid (OA)	0.019	1.820			
	Citric acid (CA)	0.32	1.694			
	Formic acid (FA)	1.29	1.631			
	EDTA acid (EDTA)	0.0034	2.847			
Substitution extraction experiments Concentration in [%]	TA	0.5	2.505	20, 40, 60	500	300
		1.0	2.168			
		2.0	1.937			
	OA	0.5	1.239			
		1.0	1.488			
		2.0	1.206			
	CA	0.5	2.368			
		1.0	2.301			
		2.0	2.130			
	FA	0.5	2.532			
		1.0	2.232			
		2.0	2.001			
	EDTA	0.05	3.542			

**Table 2:** Calculated effective acid solution concentrations and pH values.

Organic Acid	Acid Concentration [mol/dm^3^]	Acid Concentration [%]	pH Calculated	pH Measured
TA	0.20	3.0	1.823	1.728
OA	0.019	0.17	1.823	1.820
CA	0.31	5.9	1.824	1.794
FA	1.3	5.8	1.823	1.731
EDTA *	0.026	0.75	1.823	2.847 ^1^

* pH value for 0.5% EDTA solution concentration due to EDTA poor solubility in water.

**Table 3:** Substitution extraction parameters where Cr extraction yield above 50% was achieved.

Organic Acid	Acid Concentration [%]	Temperature [%]	N_t_ Loss [%]	Cr Yield [%]
TA	0.50%	60	8.0	60
TA	1.00%	60	6.9	72
TA	2.00%	60	15	72
OA	0.50%	60	72	68
OA	1.00%	40	1.1	57
OA	2.00%	40	1.8	57
OA	1.00%	60	44	79
OA	2.00%	60	85	73
CA	0.50%	60	8.3	54
CA	1.00%	60	3.9	53
CA	2.00%	60	23	63
FA	2.00%	60	0.9	50

**Table 4:** Substitution extraction parameters, where Cr extraction yield above 50% was achieved.

Method Reference	Temperature (°C)	Time	Type of Acid	Cr extraction Efficiency	Collagen Loss	Cr(VI) Monitoring
Substitution Extraction [30]	50	8 h	Citric and oxalic acids	78%	High decomposition above 60 °C	Not specified
Microwave-Assisted Extraction [28]	60	3 min	EDTA acid (3 mol/L)	99%	Not considered in this study	Not specified
Acid extraction [35]	40	12 h	Sulfuric and oxalic acid (1:1 ratio)	96%	High-purity collagen obtained	Not specified
Ultrasound-Assisted Extraction [33]	50	3 min + 3 × 3-min washing	EDTA (Cr/EDTA molar ratio 1:3)	>98%	Not specified	Not specified
Substitution extraction (this study)	43.6	5 h	Oxalic acid 1.3 wt%	63.1%	0.70%	Monitored (0.65 mg/L)

## Data Availability

Data are contained within the article or Supplementary Material.

## Supplementary Materials

The following supporting information can be downloaded at: https://www.mdpi.com/article/10.3390/ma18010118/s1, Figure S1. Pareto chart of the standardized effects; Table S1. Chromium-tanned leather shavings elemental characterization; Table S2. Quadratic model fitting summary for FA (formic acid) sample; Table S3. Quadratic model fitting summary for CA (citric acid) sample; Table S4. Quadratic model fitting summary for TA (tartaric acid) sample; Table S5. Quadratic model fitting summary for OA (oxalic acid) sample; Table S6. Linear model fitting summary for FA (formic acid) sample; Table S7. Linear model fitting summary for CA (citric acid) sample; Table S8. Linear model fitting summary for TA (tartaric acid) sample; Table S9. Linear model fitting summary for OA (oxalic acid) sample; Table S10. Quadratic model fitting results; Table S11. Linear model fitting results; Table S12. Changes in pH value during all substitution extraction experiments; Table S13. Summary of model statistics; Table S14. Analysis of variance; Table S15. Coded coefficients for process optimization.
