# Supplementary material for: Chromium Substitution Extraction Method for Its Recovery from Chromium-Tanned Leather Waste
Source: Materials (Basel). 2024 Dec 30;18(1):118. doi: 10.3390/ma18010118 (PMC11722535; doi:10.3390/ma18010118)
Supplement: Supplementary file 1 [file materials-18-00118-s001.zip › materials-3330755-supplementary.pdf]

1. Waste characterization

Table S1. Chromium-tanned leather shavings elemental characterization.

CTLS			Total solids [%]	Uncertainty [%]
			51.94	±0.24
			Volatile solids [% of TS]	Uncertainty [%]
			91.96	±0.20
No.	Element	Unit	Content (in FM*)	Uncertainty
1	N	[g/kg]	83.380	±0.090
2	P		0.30	± 0.050
3	K		0.32	± 0.050
4	S		6.5	± 0.90
5	Ca		1.72	± 0.25
6	Mg		0.534	± 0.080
7	Na		5.66	± 0.85
8	Cu	[mg/kg]	7.23	± 1.1
9	Fe		83.1	± 13
10	Mn		0.508	± 0.076
11	Mo		<LOD (<0.21)	
12	Zn		18.1	± 2.7
13	As		<LOD (<5.0)	
14	Ba		<LOD (<0.020)	
15	Cd		<LOD (<0.090)	
16	Cr	[g/kg]	17,48	± 0,10
17	Hg	[mg/kg]	<LOD (<0.0010)	
18	Ni		<LOD (<0.56)	
19	Pb		<LOD (<0.36)	
20	Al		8.9	± 1.3
21	Co		<LOD (0.27)	
22	Se		<LOD (0.49)	

* - FM – in Fresh Matter

Analytical procedure:

Elemental composition analysis, excluding nitrogen (N), was conducted using Inductively Coupled Plasma Optical Emission Spectrometry (ICP-OES) following accredited methods (EN ISO 17025). A homogeneous 0.5 g sample of CTLS was prepared by microwave digestion using 10 mL of aqua regia, which consist of a mixture of 2.5 mL nitric acid (69% Tracepure, Merck KGaA, Germany) and 7.5 mL hydrochloric acid (36% Tracepure, Merck KGaA, Germany). After digestion, the samples were filtered and diluted to a final volume of 50 mL before analysis. The ICP-OES spectrometer used for analysis was the VISTA-MPX (Varian, Australia).

Total nitrogen content was determined using the conductometric method with the CN Vario MACRO Cube elemental analyzer equipped with a Thermal Conductivity Detector (TCD) from ELEMENTAR Analysensysteme, Germany. Approximately 50 mg of CTLS samples were enclosed in tin foil capsules and introduced into the apparatus. Prepared sample was then dry combust at 900°C in the presence of gaseous oxygen and formed gases was analyzed.

2. Calculations and fitting a quadratic model to the data for all the investigated acids.

Table S2. Quadratic model fitting summary for FA (formic acid) sample.

Sample	FA (formic acid)			R-squared:		0.993
Model	OLS			Adj. R-squared		0.982
Method	Least Squares			F-statistic:		87.25
Observations	9			Prob (F-statistic):		0.00190
				Log-Likelihood:		-16.319
	**coef**	**std err**	**t**	**P>|t|**	**[0.025**	**0.975]**
β_0	5.8721	9.009	0.652	0.561	-22.8	34.544
β_1	-0.9584	0.38	-2.522	0.086	-2.168	0.251
β_2	8.3493	10.185	0.82	0.472	-24.064	40.762
β_3	0.0222	0.005	4.878	0.016	0.008	0.037
β_4	0.188	0.084	2.236	0.111	-0.08	0.456
β_5	-3.8936	3.7	-1.052	0.37	-15.669	7.882
Omnibus:		1.443		Durbin-Watson:	2.454	
Prob(Omnibus):		0.486		Jarque-Bera (JB):	0.956	
Skew:		0.561		Prob(JB):	0.620	
Kurtosis:		1.865		Cond. No.:	3.41e+04	

Table S3. Quadratic model fitting summary for CA (citric acid) sample.

Sample	CA (citric acid)			R-squared:		0.963
Model	OLS			Adj. R-squared		0.902
Method	Least Squares			F-statistic:		15.74
Observations	9			Prob (F-statistic):		0.0231
				Log-Likelihood:		-25.146
	**coef**	**std err**	**t**	**P>|t|**	**[0.025**	**0.975]**
β_0	15.751	24.022	0.656	0.559	-60.698	92.2
β_1	-0.1324	1.013	-0.131	0.904	-3.357	3.092
β_2	-28.4195	27.156	-1.047	0.372	-114.843	58.004
β_3	0.0153	0.012	1.265	0.295	-0.023	0.054
β_4	0.0879	0.224	0.392	0.721	-0.626	0.802
β_5	11.8987	9.866	1.206	0.314	-19.499	43.296
Omnibus:		0.132		Durbin-Watson:	3.097	
Prob(Omnibus):		0.936		Jarque-Bera (JB):	0.286	
Skew:		-0.206		Prob(JB):	0.867	
Kurtosis:		2.229		Cond. No.:	3.41e+04	

Table S4. Quadratic model fitting summary for TA (tartaric acid) sample.

Sample	TA (tartaric acid)			R-squared:		0.996
Model	OLS			Adj. R-squared		0.988
Method	Least Squares			F-statistic:		136.2
Observations	9			Prob (F-statistic):		0.000982
				Log-Likelihood:		-16.206
	**coef**	**std err**	**t**		**coef**	**std err**
β_0	-20.9317	8.896	-2.353	β_0	-20.9317	8.896
β_1	0.9641	0.375	2.569	β_1	0.9641	0.375
β_2	24.4265	10.057	2.429	β_2	24.4265	10.057
β_3	0.0034	0.004	0.766	β_3	0.0034	0.004
β_4	0.0756	0.083	0.911	β_4	0.0756	0.083
β_5	-8.4801	3.654	-2.321	β_5	-8.4801	3.654
Omnibus:		0.002		Durbin-Watson:	2.931	
Prob(Omnibus):		0.999		Jarque-Bera (JB):	0.213	
Skew:		0.015		Prob(JB):	0.899	
Kurtosis:		2.246		Cond. No.:	3.41e+04	

Table S5. Quadratic model fitting summary for OA (oxalic acid) sample.

Sample	OA (oxalic acid)			R-squared:		0.990
Model	OLS			Adj. R-squared		0.974
Method	Least Squares			F-statistic:		60.17
Observations	9			Prob (F-statistic):		0.0033
				Log-Likelihood:		-20.233
	**coef**	**std err**	**t**		**coef**	**std err**
β_0	-56.6003	13.917	-4.067	β_0	-56.6003	13.917
β_1	2.746	0.587	4.678	β_1	2.746	0.587
β_2	45.0706	15.733	2.865	β_2	45.0706	15.733
β_3	-0.0153	0.007	-2.185	β_3	-0.0153	0.007
β_4	-0.1378	0.13	-1.061	β_4	-0.1378	0.13
β_5	-12.9662	5.716	-2.269	β_5	-12.9662	5.716
Omnibus:		0.523		Durbin-Watson:	2.201	
Prob(Omnibus):		0.77		Jarque-Bera (JB):	0.532	
Skew:		-0.319		Prob(JB):	0.767	
Kurtosis:		1.994		Cond. No.:	3.41e+04	

3. **Calculations and fitting a linear model to the data for all the investigated acids.**

Table S6. Linear model fitting summary for FA (formic acid) sample.

Sample	FA (formi acid)			R-squared:		0.925
Model	OLS			Adj. R-squared		0.900
Method	Least Squares			F-statistic:		37.05
Observations	9			Prob (F-statistic):		0.00042
				Log-Likelihood:		-27.096
	**coef**	**std err**	**t**		**coef**	**std err**
β_0	-27.5738	6.498	-4.243	β_0	-27.5738	6.498
β_1	1.0332	0.123	8.414	β_1	1.0332	0.123
β_2	5.8577	3.216	1.822	β_2	5.8577	3.216
Omnibus:	2.878	Durbin-Watson:	2.875	Omnibus:	2.878	Durbin-Watson:
Prob(Omnibus):	0.237	Jarque-Bera (JB):	0.92	Prob(Omnibus):	0.237	Jarque-Bera (JB):
Skew:	-0.098	Prob(JB):	0.631	Skew:	-0.098	Prob(JB):
Kurtosis:		1.446		Cond. No.:	147	
Sample		FA (formi acid)		R-squared:	0.925	
Model		OLS		Adj. R-squared	0.900	
Method		Least Squares		F-statistic:	37.05	

Table S7. Linear model fitting summary for CA (citric acid) sample.

Sample	CA (citric acid)			R-squared:		0.924
Model	OLS			Adj. R-squared		0.899
Method	Least Squares			F-statistic:		36.48
Observations	9			Prob (F-statistic):		0.000439
				Log-Likelihood:		-28.418
	**coef**	**std err**	**t**		**coef**	**std err**
β_0	-23.6431	7.526	-3.141	β_0	-23.6431	7.526
β_1	1.1953	0.142	8.404	β_1	1.1953	0.142
β_2	5.6929	3.724	1.528	β_2	5.6929	3.724
Omnibus:	18.277	Durbin-Watson:	2.116	Omnibus:	18.277	Durbin-Watson:
Prob(Omnibus):	0	Jarque-Bera (JB):	8.925	Prob(Omnibus):	0	Jarque-Bera (JB):
Skew:	-2.001	Prob(JB):	0.0115	Skew:	-2.001	Prob(JB):
Kurtosis:		5.789		Cond. No.:	147	
Sample		CA (citric acid)		R-squared:	0.924	
Model		OLS		Adj. R-squared	0.899	
Method		Least Squares		F-statistic:	36.48	

Table S8. Linear model fitting summary for TA (tartaric acid) sample.

Sample	TA (tartaric acid)			R-squared:		0.986
Model	OLS			Adj. R-squared		0.981
Method	Least Squares			F-statistic:		206.3
Observations	9			Prob (F-statistic):		2.94E-06
				Log-Likelihood:		-21.534
	**coef**	**std err**	**t**		**coef**	**std err**
β_0	-27.5738	6.498	-4.243	β_0	-27.5738	6.498
β_1	1.0332	0.123	8.414	β_1	1.0332	0.123
β_2	5.8577	3.216	1.822	β_2	5.8577	3.216
Omnibus:	2.516	Durbin-Watson:	2.706	Omnibus:	2.516	Durbin-Watson:
Prob(Omnibus):	0.284	Jarque-Bera (JB):	0.928	Prob(Omnibus):	0.284	Jarque-Bera (JB):
Skew:	0.786	Prob(JB):	0.629	Skew:	0.786	Prob(JB):
Kurtosis:		2.918		Cond. No.:	147	
Sample		TA (tartaric acid)		R-squared:	0.986	
Model		OLS		Adj. R-squared	0.981	
Method		Least Squares		F-statistic:	206.3	

Table S9. Linear model fitting summary for OA (oxalic acid) sample.

Sample	FA (tartaric acid)			R-squared:		0.954
Model	OLS			Adj. R-squared		0.938
Method	Least Squares			F-statistic:		61.89
Observations	9			Prob (F-statistic):		9.88E-05
				Log-Likelihood:		-27.096
	**coef**	**std err**	**t**		**coef**	**std err**
β_0	-13.5168	6.559	-2.061	β_0	-13.5168	6.559
β_1	1.3586	0.124	10.96	β_1	1.3586	0.124
β_2	6.2158	3.246	1.915	β_2	6.2158	3.246
Omnibus:	1.946	Durbin-Watson:	1.627	Omnibus:	1.946	Durbin-Watson:
Prob(Omnibus):	0.378	Jarque-Bera (JB):	0.803	Prob(Omnibus):	0.378	Jarque-Bera (JB):
Skew:	0.721	Prob(JB):	0.669	Skew:	0.721	Prob(JB):
Kurtosis:		2.749		Cond. No.:	147	
Sample		FA (tartaric acid)		R-squared:	0.954	
Model		OLS		Adj. R-squared	0.938	
Method		Least Squares		F-statistic:	61.89	

Table S10. Quadratic model fitting results.

Sample	Equation
FA	$Cr_{yield} = 5.8721 - 0.9584 \cdot T + 8.3493 \cdot C_{FA} + 0.0222 \cdot T^2 + 0.188 \cdot C_{FA}^2 - 3.8936 \cdot TC_{FA}$
CA	$Cr_{yield} = 15.751 - 0.1324 \cdot T - 28.4195 \cdot C_{CA} + 0.0153 \cdot T^2 + 0.0879 \cdot C_{CA}^2 + 11.8987 \cdot TC_{CA}$
TA	$Cr_{yield} = -20.9317 + 0.9641 \cdot T + 24.4265 \cdot C_{TA} + 0.0034 \cdot T^2 + 0.0756 \cdot C_{TA}^2 - 8.4801 \cdot TC_{TA}$
OA	$Cr_{yield} = -56.6003 + 2.746 \cdot T + 45.0706 \cdot C_{OA} - 0.0153 \cdot T^2 - 0.1378 \cdot C_{OA}^2 - 12.9662 \cdot TC_{OA}$

Table S11. Linear model fitting results.

Sample	Equation
FA	$Cr_{yield} = -27.5738 + 1.0332 \cdot T + 5.8577 \cdot C_{FA}$
CA	$Cr_{yield} = -23.6431 + 1.1953 \cdot T + 5.6929 \cdot C_{CA}$
TA	$Cr_{yield} = -18.4425 + 1.3272 \cdot T + 5.6457 \cdot C_{TA}$
OA	$Cr_{yield} = -27.5738 + 1.3586 \cdot T + 6.2158 \cdot C_{OA}$

Table S12. Changes in pH value during all substitution extraction experiments.

	0,50%				1,00%				2,00%			
	T [°C]	pH_0	pH_{end}	Cr yeild [%]	T [°C]	pH_0	pH_{end}	Cr yeild [%]	T [°C]	pH_0	pH_{end}	Cr yeild [%]
FA	20	2,432	2,498	1,9	20	2,032	2,175	2,9	20	2,001	2,001	3,9
	40		2,500	10	40		2,093	13	40		2,009	21
	60		2,560	36	60		2,071	47	60		2,051	50
CA	20	2,268	2,274	5,5	20	2,201	2,212	11	20	2,030	2,093	11
	40		2,265	29	40		2,516	15	40		2,114	37
	60		2,505	54	60		2,502	53	60		2,091	63
TA	20	2,405	2,461	11	20	2,168	2,180	17	20	1,907	1,931	18
	40		2,477	36	40		2,222	40	40		1,991	45
	60		2,498	60	60		2,231	72	60		1,908	72
OA	20	1,239	1,248	14	20	1,228	1,397	18	20	1,206	1,270	25
	40		1,264	42	40		1,377	57	40		1,223	57
	60		1,250	68	60		1,256	79	60		1,278	73

Table S13. Summary of model statistics.

S	R-sq	R-sq(adj)	R-sq(pred)
3,96880	99,01%	97,37%	90,35%

Table S14. Analysis of Variance.

Source	DF	Adj SS	Adj MS	F-Value	P-Value
Model	5	4738,93	947,79	60,17	0,003
Linear	2	4450,84	2225,42	141,28	0,001
Temperature [°C]	1	4278,69	4278,69	271,64	0,000
Concentration [%]	1	172,15	172,15	10,93	0,046
Square	2	156,29	78,14	4,96	0,112
Temperature [°C]*Temperature [°C]	1	75,23	75,23	4,78	0,117
Concentration [%]*Concentration [%]	1	81,06	81,06	5,15	0,108
2-Way Interaction	1	17,73	17,73	1,13	0,367
Temperature [°C]*Concentration [%]	1	17,73	17,73	1,13	0,367
Error	3	47,25	15,75		
Total	8	4786,18			

Table S15. Coded Coefficients for Process Optimization.

Term	Coef	SE Coef	T-Value	P-Value	VIF
Constant	57,89	3,25	17,82	0,000	
Temperature [°C]	26,94	1,63	16,48	0,000	1,02
Concentration [%]	5,36	1,62	3,31	0,046	1,04
Temperature [°C]*Temperature [°C]	-6,13	2,81	-2,19	0,117	1,00
Concentration [%]*Concentration [%]	-7,29	3,22	-2,27	0,108	1,04
Temperature [°C]*Concentration [%]	-2,07	1,95	-1,06	0,367	1,02

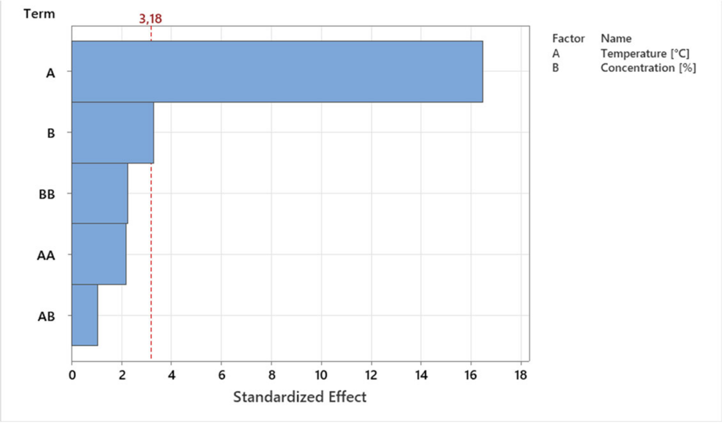

Figure S1. Pareto chart of the standardized effects.
